# Attachment Patterns of Avian Influenza H5 Clade 2.3.4.4b Virus in Respiratory Tracts of Marine Mammals, North Atlantic Ocean

**DOI:** 10.3201/eid3109.250499

**Published:** 2025-09

**Authors:** Syriam Sooksawasdi Na Ayudhya, Lonneke Leijten, Willemijn F. Rijnink, Monique I. Spronken, Thijs Kuiken, Lisa Bauer, Debby van Riel

**Affiliations:** Prince of Songkla University, Faculty of Veterinary Science, Songkhla, Thailand (S. Sooksawasdi Na Ayudhya); Erasmus University Rotterdam, Erasmus Medical Center, Rotterdam, the Netherlands (S. Sooksawasdi Na Ayudhya, L. Leijten, W.F. Rijnink, M.I. Spronken, T. Kuiken, L. Bauer, D. van Riel)

**Keywords:** influenza, influenza A virus, highly pathogenic avian influenza virus, H5N1, respiratory infections, zoonoses, viruses, virus attachment, virus histochemistry, respiratory tract, harbor seals, grey seals, harbor porpoise, bottlenose dolphin, North Atlantic Ocean, cetaceans, pinnipeds

## Abstract

Highly pathogenic avian influenza A(H5N1) clade 2.3.4.4b virus infections have caused substantial mortality events in marine mammals in recent years. We hypothesized that the high number of infections and disease severity could be related to cell tropism in respiratory tracts. Therefore, we examined the attachment pattern of an H5N1 clade 2.3.4.4b virus (H5^2022^) as a measure for cell tropism in the respiratory tracts of harbor seals, gray seals, harbor porpoises, and bottlenose dolphins and compared it with an H5N1 clade 2.1.3.2 virus (H5^2005^) and a human seasonal H3N2 virus using virus histochemistry. Both H5 viruses attached abundantly to olfactory and respiratory mucosa in the upper respiratory tract of both seal species. H5^2022^ attached more abundantly than H5^2005^ to epithelial cells in the lower respiratory tract of all species. The observed attachment possibly explains the susceptibility of marine mammal species for recent H5N1 viruses and the observed development of severe disease.

Highly pathogenic avian influenza (HPAI) H5Nx virus of the A/goose/Guangdong/1/1996 lineage was first detected in domestic geese in 1996 and has since infected predominantly poultry (*1*). Since 2020, H5Nx viruses have circulated in wild birds and have spread almost worldwide (*2*). Viruses from the A/goose/Guangdong/1/1996 lineage pose a substantial threat to wild and endangered species (*3*,*4*), domestic species (*5*), and humans (*6*). H5N1 viruses of clade 2.3.4.4b are circulating in wild birds in Eurasia (*4*) North America (*7*), South America (*8*), Southern Africa (*9*), and Antarctica (*10*). Circulation has been associated with transmission to and outbreaks in marine mammals (*8*,*11*,*12*).

Historically, multiple mortality events in marine mammals have been linked to avian influenza A viruses. In different seal species, infections with H7N7 viruses in 1979–1980 (*13*), H4N5 viruses in 1982–1983 (*14*), H4N6 viruses in 1991, H3N3 viruses in 1992 (*15*), H10N7 viruses in 2014 (*16*), and H5N8 viruses in 2016, 2017 (*17*), and 2021 (*18*,*19*) have been reported. In addition, serologic evidence for infections with human 2009 pandemic influenza A(H1N1) virus was detected in northern elephant seals (*Mirounga angustirostris*), harbor seals (*Phoca vitulina*), and California sea lions (*Zalophus californianus*) (*20*). The currently circulating HPAI H5Nx clade 2.3.4.4b viruses have infected many marine mammal species from phylogenetically different families (Appendix Figure 1). HPAI H5Nx viruses have caused mortality events in different pinniped species, such as harbor seals (*21*,*22*), gray seals (*Halichoerus grypus*) (*8*,*23*,*24*), walruses (*Odobenus rosmarus*) (*4*,*25*), and elephant seals (*Mirounga leonina*) (*3*). In addition, HPAI H5N1 virus caused mass dieoffs in sea lions (*Otaria flavescens*) on the Pacific coast of South America (*8*,*11*). Cetacean species were also found to be infected with H5Nx clade 2.3.4.4b viruses; respiratory or neurologic disease has been reported in 3 common dolphins (*Delphinus delphis*) in Peru, Wales, and England (*8*); 2 harbor porpoises (*Phocoena phocoena*) in Sweden and England (*26*); an Atlantic white-sided dolphin (*Lagenorhynchus acutus*) in Canada (*27*); and a common bottlenose dolphin (*Tursiops truncatus*) in Florida, USA (*28*).

The receptor binding of influenza A virus to sialic acid moieties on glycoproteins and glycolipids, and the distribution of those receptors in a host, are critical determinants for host range and cell tropism (*29*). Human and avian influenza A viruses vary in their sialic acid‐binding preference. Simplified, avian influenza A viruses preferentially bind to α2,3-linked sialic acids, whereas human influenza A viruses prefer α2,6-linked sialic acids (*30*,*31*). Knowledge of sialic acid expression in marine mammals is limited to harbor seals showing the presence of α2,3- and α2,6-linked sialic acids in the lower respiratory tract (*32*). Studying the direct binding of influenza A viruses to marine mammal respiratory tissues, using virus histochemistry, has revealed that human seasonal influenza H3N2 or H1N1 viruses rarely attach to the trachea and bronchi of seals and cetaceans (*33*). In contrast, low pathogenicity avian influenza A viruses H4N5 and H7N7 do attach to trachea and bronchi of harbor and gray seals but not to the trachea and bronchi of cetaceans (*33*). Both human and avian influenza A viruses attach to bronchiolar and alveolar epithelial cells in seals and cetaceans (*33*).

The ability of HPAI H5N1 clade 2.3.4.4b viruses to infect and cause severe disease in a broad range of mammal species has not been previously observed with other avian influenza A viruses (*3*,*34*). The attachment pattern in the respiratory tract of marine mammals of H5N1 clade 2.3.4.4b virus, and whether that pattern differs from the attachment pattern of previously circulating H5 viruses from different clades, is unknown. Therefore, we compared the attachment pattern of a 2022 H5N1 clade 2.3.4.4b virus, a 2005 H5N1 clade 2.1.3.2 virus, and a seasonal human H3N2 virus in the respiratory tracts of marine mammals commonly found in the North Atlantic Ocean: harbor seals, gray seals, harbor porpoises, and bottlenose dolphins.

## Material and Methods

### Cells

For testing, we used human epithelial kidney 293T cells and Madin-Darby canine kidney (MDCK) cells. We maintained human epithelial kidney 293T cells (ATCC accession no. ATCC-CRL-3216) in Dulbecco’s Modified Eagle Medium (Capricorn Scientific, https://www.capricorn-scientific.com) supplemented with 1 mmol sodium pyruvate (Thermo Fisher Scientific, https://www.thermofisher.com), 100 IU/mL penicillin, 100 µg/µL streptomycin, 2 mmol glutamine, 1× nonessential amino acids (all Capricorn Scientific), 500 µg/mL geneticin (Thermo Fisher Scientific), and 10% fetal bovine serum at 37°C and 5% CO_2_. We maintained MDCK cells in Eagle’s minimum essential medium (Capricorn Scientific) supplemented with 1.5 mg/mL sodium bicarbonate (Thermo Fisher Scientific), 10 mmol HEPES buffering agent, 100 IU/mL penicillin, 100 µg/µL streptomycin, 2 mmol glutamine, 1× nonessential amino acids (all Capricorn Scientific), and 10% fetal bovine serum.

### Recombinant Viruses, Virus Preparation, Inactivation, and Labeling for Virus Histochemistry

To work at Biosafety Level 2, we performed virus histochemistry with recombinant viruses that contain 7 segments of the mouse-adapted influenza A virus strain A/Puerto Rico/8/1934 (A/PR/8/34) and the hemagglutinin (HA) segment of either H5 (A/Indonesia/05/2005 [H5N1]; referred to as H5^ΔMBCS2005^) (*35*) from clade 2.1.3.2, H5 (A/Caspian gull/Netherlands/1/2022 [H5N1]; referred to as H5^ΔMBCS2022^) from clade 2.3.4.4b, or H3 (A/Netherlands/213/2003 [H3N2]; referred to as H3^2003^), generated as described previously (*36*). We performed site-directed mutagenesis with the Pfu Ultra II Fusion HS DNA Polymerase (Agilent, https://www.agilent.com) and specific primers to remove the multibasic cleavage site (MBCS), which we replaced with the conserved H5 low pathogenic cleavage site, as described previously (*36*). One day before transfection, we seeded 293T cells and subsequently transfected with 5 µg of the desired HA gene segment and 5 µg of each of the remaining A/PR/8/34 gene segments. Approximately 16 hours after transfection, we removed the supernatant and washed the cells with phosphate-buffered saline (PBS) once. Three days after transfection, we harvested supernatant and passaged the virus once on a confluent layer of MDCK cells. We determined the presence of virus 3 days after inoculation with an hemagglutination assay to determine the hemagglutination units (HAU).

We inoculated MDCK cells with the described influenza A viruses at 10^−3^, 10^−4^, and 10^−5^ dilution. Three days after inoculation, we harvested the supernatant with the highest HAU and cleared it by low-speed centrifugation. We subsequently centrifuged cleared supernatants for 2 hours at 27,000 rpm in a SW32 rotor (Beckman Coulter, https://www.beckman.com) at 4°C on a 0.5 mL layer of 60% sucrose. We transferred the lowermost 2.5-mL virus supernatant on top of the sucrose cushion to a 60%–20% sucrose gradient and centrifuged overnight at 32,000 rpm in a SW41 rotor (Beckman Coulter,) at 4°C. We harvested the virus fraction, diluted in PBS, and centrifuged for 2 hours at 27,000 rpm in a SW32 rotor (Beckman Coulter) at 4°C to deplete leftover sucrose. We then resuspended the virus pellet in PBS and inactivated by dialyzing against 0.1% formalin for 3 days at room temperature. We labeled virus by mixing with an equal volume of 0.1 mg/mL of fluorescein isothiocyanate (FITC) (Sigma-Aldrich, https://www.sigmaaldrich.com) in 0.5 mol bicarbonate buffer (pH 9.5) for 1 hour at room temperature while constantly stirring. To lose all unbound FITC, we dialyzed labeled virus against PBS, then determined the HAU.

### Hemagglutination Assay

We serially diluted viruses (1:2 dilution) in 0.1 mol PBS at pH 7.2. We mixed 50 µL of diluted virus with 50 µL of 0.5% turkey erythrocytes in a U-bottom plate and incubated for 1 hour at 4°C. We read the titer of each isolate as the reciprocal of the highest dilution in which complete hemagglutination was observed and recorded as the HAU per 25 µL.

### Marine Mammal Tissue Testing

We tested formalin-fixed, paraffin-embedded (FFPE) tissues of the upper respiratory tract (nasal turbinate) from 2 harbor seals and 1 gray seal, and lower respiratory tract (trachea, bronchus, bronchiole, and alveoli) from 3 harbor seals, 3 gray seals, 3 harbor porpoises, and 3 bottlenose dolphins. The exact ages of the animals were unknown, but during necropsy, harbor and gray seals were reported as juvenile or subadult, harbor porpoises as neonate or juvenile, and bottlenose dolphins as neonate or adult. We included >1 slide of each tissue in every staining. Harbor seal, gray seal, and harbor porpoise FFPE tissues were derived from the Erasmus MC FFPE tissues archive, and bottlenose dolphin FFPE tissues were kindly provided by Dr. Toni Ramis. Selected tissues showed no abnormalities or histologic lesions.

### Virus Histochemistry Staining on Marine Mammal Respiratory Tissues and Scoring

We deparaffinized 3-μm thick FFPE tissue sections with xylene and rehydrated using graded ethanol. We incubated slides overnight at 4°C with 100 µL FITC-labeled influenza virus (50 HAU). For visualization by light microscopy (Olympus, https://www.olympus-global.com), we detected the FITC label with a peroxidase-labeled rabbit anti-FITC antibody (Agilent). We amplified the signal with a tyramide signal amplification kit (Perkin Elmer, https://www.perkinelmer.com), according to manufacturer instructions. Peroxidase was revealed with 3-amino-9-ethylcarbazole (Sigma-Aldrich), resulting in a bright red precipitate. We counterstained tissues with hematoxylin and embedded in glycerol-gelatin (Merck, https://www.merck.com). For the negative control, we omitted the FITC-labeled virus; for the positive control, we used FFPE tissue sections of the ferret respiratory tract for H3 virus and of duck colon for H5 virus.

We scored the mean abundance of cells to which virus attached to the apical side from each individual tissue section of each marine mammal species as follows: −, no attachment; +, attachment to rare or few cells (<10% cells positive); +, attachment to a moderate number of cells (10%–50% cells positive); and ++, attachment to many cells (>50% cells positive). Overall, we found little variation in the scores between individual animals, and we recorded the median score for each species (Table). Where possible, we recorded the predominant cell type to which virus attached: ciliated epithelial cell, goblet cell, or alveolar epithelial cell. In addition, we scored if viruses attached intracellular to epithelial cells of the submucosal gland or Bowman’s gland. We took images of virus attachment from a representative tissue section of each animal species at an original magnification of ×1,000.

**Table:** Attachment of 2 HPAI viruses and 1 human seasonal influenza virus in study of attachment patterns of avian influenza H5 clade 2.3.4.4b virus in respiratory tracts of marine mammals, North Atlantic Ocean*

Tissue	Species	Seasonal H3^2003^			HPAI H5^ΔMBCS2005^			HPAI H5^ΔMBCS2022^	
		Score	Preferred cell type		Score	Preferred cell type		Score	Preferred cell type
Olfactory mucosa	Harbor seal	++	cil, bg		++	cil, bg		++	cil, bg
	Gray seal	+	cil, bg		++	cil, bg		++	cil, bg
Respiratory mucosa	Harbor seal	+	cil, sg		++	cil, sg		++	cil, sg
	Gray seal	+	cil		++	cil, gb		++	cil, gb
Trachea	Harbor seal	+	cil		+	cil		+	cil
	Gray seal	+	cil		+	cil, sg		++	cil, sg
	Harbor porpoise	+	cil		–	cil		+	cil, sg
	Bottlenose dolphin	+	cil, sg		–			–	
Bronchus	Harbor seal	–			±	cil sg		++	cil, sg
	Gray seal	–			++	cil sg		++	cil, sg
	Harbor porpoise	+	cil		–			+	cil
	Bottlenose dolphin	+	cil		–			–	
Bronchiole	Harbor seal	–			+	cil		+	cil
	Gray seal	–			+	cil, sg		+	cil, sg
	Harbor porpoise	++	cil		+	cil		+	cil
	Bottlenose dolphin	++	cil		+	cil		+	cil
Alveoli	Harbor seal	+	aec		+	aec		+	aec
	Gray seal	+	aec		+	aec		+	aec
	Harbor porpoise	++	aec		+	aec		++	aec
	Bottlenose dolphin	++	aec		+	aec		+	aec

*Scoring indicates attachment of viruses to the apical side of ciliated cells of the olfactory mucosa, respiratory mucosa, trachea, bronchus, bronchiole, and alveoli of marine mammal tissue sections. aec, alveolar epithelial cell; bg, Bowman’s gland; cil, ciliated epithelial cell; gb, goblet cell; H3^2003^, A/Netherlands/213/2003 (H3N2); H5^ΔMBCS2005^, A/Indonesia/05/2005 (H5N1); H5^ΔMBCS2022^, A/Caspian gull/Netherlands/1/2022 (H5N1); HPAI, highly pathogenic avian influenza; sg, submucosal gland; ++, abundant (>50% cells positive); +, moderate (10%–50% cells positive); ±, scarce (<10% cells positive); –, negative (no attachment).

### Phylogenetic Analysis of Marine Mammals

We obtained complete genome sequences of marine mammal species infected by H5 virus clade 2.3.4.4b, according to the list of the European Food Safety Authority report (*27*), from National Center for Biotechnology Information taxonomy database and GenBank. We aligned sequences with ClustalW (http://www.clustal.org) using MEGA version 11 (https://www.megasoftware.net) and transformed to PHYLIP in ALTER (https://phylipweb.github.io/phylip). We constructed a maximum-likelihood tree in FigTree version 1.4.4 (http://tree.bio.ed.ac.uk/software/figtree) using RAxMLHPC2 (https://www.phylo.org/tools/raxmlhpc2_tgb.html) on ACESS version 8.2.12 with rapid bootstrapping run on XSDE (*37*).

### Consensus HA Sequence of H5N1 Clade 2.3.4.4.b Viruses Found in Marine Mammals

We downloaded all available influenza A H5 HA nucleotide sequences and accompanying metadata from GISAID (https://www.gisaid.org) (*38*) (Appendix). We aligned HA sequences by using Clustal Omega 22 (https://www.ebi.ac.uk/jdispatcher/msa/clustalo?stype=protein) (*39*) and trimmed the alignment to the start and stop codons of the HA sequences. After alignment, we built a consensus sequence of the influenza A H5 HA of all marine mammals and used it for alignment with other H5N1 viruses. We aligned the sequences with Clustal Omega 22 (*39*) and analyzed sequence similarities and secondary structure information with ESPRIPT version 3.023 (*40*).

## Results

### Attachment Pattern of H5^ΔMBCS2022^ and H5^ΔMBCS2005^ to Nasal Turbinate of Seals

To determine whether the HA of A/Caspian gull/Netherlands/1/2022 is similar to other H5N1 viruses from clade 2.3.4.4b detected in marine mammals, we generated a consensus sequence of all available marine mammal influenza A H5 HA sequences. An alignment of the HA of A/Caspian gull/Netherlands/1/2022 to a marine mammal consensus sequence (Appendix) showed several amino acid differences: D88G, M104L, Q115L, A210V, V510I, and M532I (H5 numbering) (Appendix Figure 2). Those amino acids are not in or close to the receptor-binding site or in amino acid residues known to affect receptor binding (*41*). Whether the amino acid changes result in differences in receptor binding properties or how they affect HA protein stability is unknown. A comparison of the HA sequence of A/Caspian Gull/Netherlands/1/2022 with H5N1 clade 2.1.3.2 virus A/Indonesia/5/2005 revealed amino acid changes known to affect receptor binding, at positions 133, 155, 156, 189 and 225 (H5 numbering) (Appendix Figure 2) (*41*,*42*; L. Bauer et al., unpub. data, https://www.biorxiv.org/content/10.1101/2024.11.27.625596v1.full).

**Figure 1:**
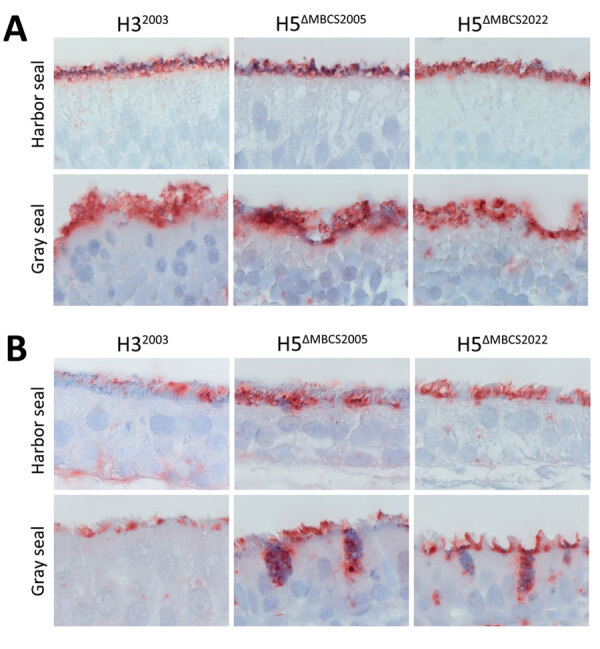
Attachment of influenza A viruses to the olfactory mucosa and respiratory mucosa of seals in study of attachment patterns of avian influenza H5 clade 2.3.4.4b virus in respiratory tracts of marine mammals, North Atlantic Ocean. Hematoxylin and eosin stain (red) shows attachment of human seasonal influenza A virus H3^2003^ and avian influenza A viruses H5^ΔMBCS2005^ and H5^ΔMBCS2022^ to the olfactory (A) and respiratory (B) mucosa in the nasal turbinate of harbor seals (*Phoca vitulina*) and gray seals (*Halichoerus grypus*). Photos were taken at high magnification (×1,000) of the apical side of the mucosa; for this reason, Bowman’s glands in the submucosa are not represented. H3^2003^, A/Netherlands/213/2003 (H3N2); H5^ΔMBCS2005^, A/Indonesia/05/2005 (H5N1); H5^ΔMBCS2022^, A/Caspian gull/Netherlands/1/2022 (H5N1).

Because of the differences in anatomy between pinnipeds and cetaceans, the nasal turbinate is absent in cetaceans (*43*). In the nasal turbinates of harbor seals and gray seals, the attachment pattern of H5^ΔMBCS2022^, H5^ΔMBCS2005^, and H3^2003^ revealed that all 3 viruses attached abundantly to the apical side of the olfactory mucosa and to the Bowman’s glands (Table; Figure 1). In the respiratory mucosa, all viruses attached predominantly to the apical side of ciliated epithelial cells. In general, both H5 viruses attached to the respiratory mucosa more abundantly than the human H3^2003^ virus (Table; Figure 1). In addition, all viruses attached to submucosal glands in the submucosa in harbor seals and both H5 viruses to goblet cells in the gray seals. Taken together, those findings indicate that both human and avian viruses can attach to upper respiratory tract tissues in harbor and gray seals and that the attachment pattern of viruses with the H5^ΔMBCS2022^ or H5^ΔMBCS2005^ are not different.

### Attachment of H5^ΔMBCS2022^ and H5^ΔMBCS2005^ to Lower Respiratory Tract Tissues 

**Figure 2:**
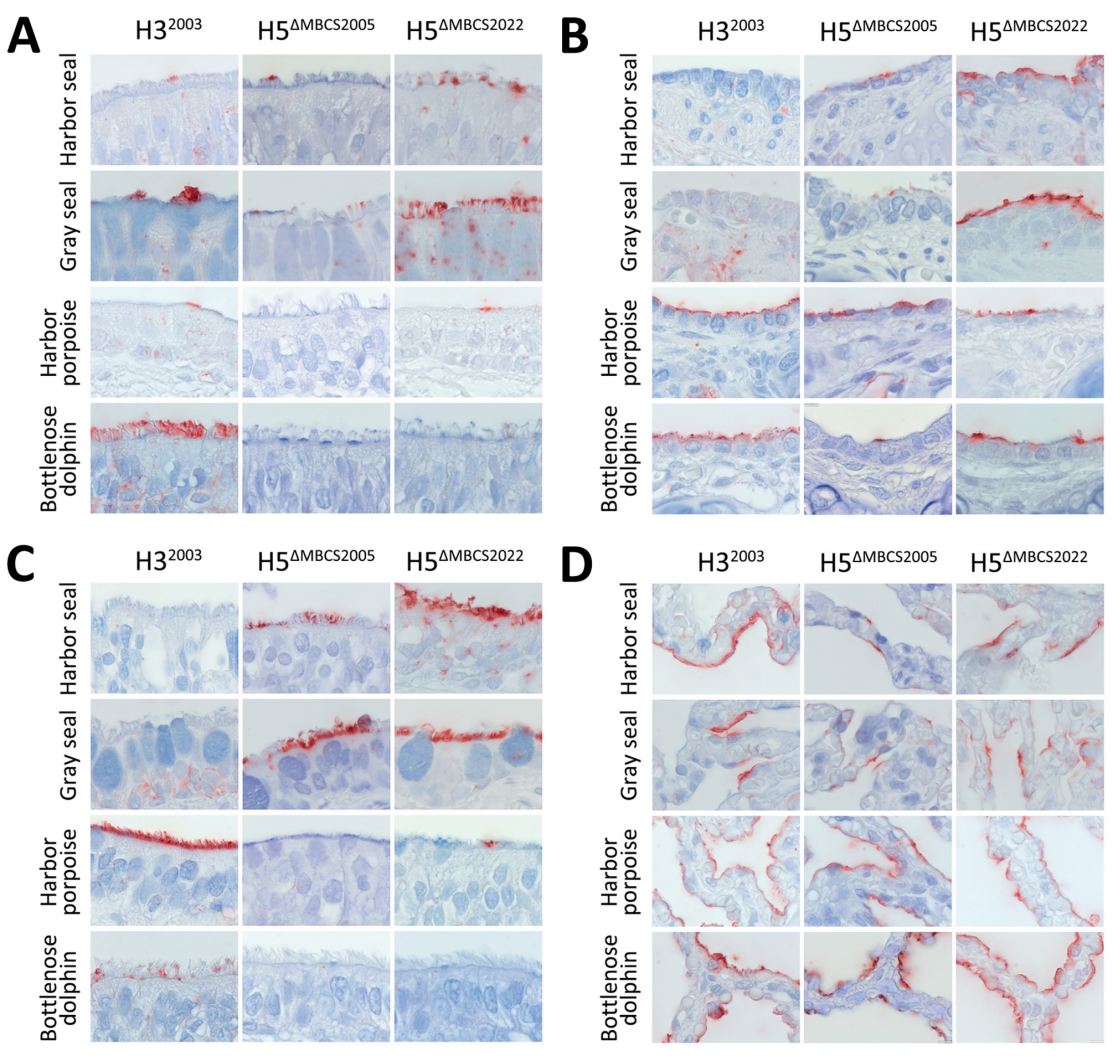
Attachment of influenza A viruses to epithelial cells of trachea, bronchus, bronchiole, and alveoli in study of attachment patterns of avian influenza H5 clade 2.3.4.4b virus in respiratory tracts of marine mammals, North Atlantic Ocean. Hematoxylin and eosin stain (red) shows attachment of human seasonal influenza A virus H3^2003^ and avian influenza A viruses H5^ΔMBCS2005^ and H5^ΔMBCS2022^ to the lower respiratory tracts of harbor seals (*Phoca vitulina*), gray seals (*Halichoerus grypus*), harbor porpoises (*Phocoena phocoena*), and bottlenose dolphins (*Tursiops truncatus*). A) Trachea; B) bronchiole; C) bronchus; D) alveoli. Photos were taken at high magnification (×1,000) of the apical side of the mucosa. H3^2003^, A/Netherlands/213/2003 (H3N2); H5^ΔMBCS2005^, A/Indonesia/05/2005 (H5N1); H5^ΔMBCS2022^, A/Caspian gull/Netherlands/1/2022 (H5N1).

In the trachea of all animals tested, all viruses attached to ciliated epithelial cells, but to varying degrees (Table). In harbor seals, gray seals, and harbor porpoises, H5^ΔMBCS2022^ attached more abundantly than H5^ΔMBCS2005^ and H3^2003^. In contrast, in bottlenose dolphins, H3^2003^ attached to a moderate number of cells, whereas we observed no detectable attachment for H5^ΔMBCS2022^ and H5^ΔMBCS2005^ (Table; Figure 2). We observed attachment to submucosal glands with H5^ΔMBCS2022^ in gray seals and harbor porpoises, with H5^ΔMBCS2005^ in harbor porpoises, and with H3^2003^ in bottlenose dolphins (Table).

In the bronchus of harbor and gray seals, both H5^ΔMBCS2022^ and H5^ΔMBCS2005^ attached to ciliated epithelial cells (Table; Figure 2) and submucosal glands (Table), whereas we did not observe any attachment with H3^2003^. In the bronchus of both cetaceans, H3^2003^ attached to a moderate number of cells. For H5 viruses, we only observed attachment with H5^ΔMBCS2022^ in the bronchus of harbor porpoises (Table; Figure 2).

In the bronchiole of all species, both H5 viruses attached to ciliated epithelial cells. In general, the attachment of H5^ΔMBCS2022^ was more abundant than that of H5^ΔMBCS2005^. Human H3^2003^ did not attach the bronchiole of harbor and gray seals, whereas it attached abundantly to the ciliated epithelial cells in the cetaceans (Table; Figure 2).

In the alveoli, all viruses attached to alveolar epithelial cells but with different abundance among different species. In harbor and gray seals, H5^ΔMBCS2022^ attached more abundantly than H5^ΔMBCS2005^ and H3^2003^. In contrast, in harbor porpoises, both H5^ΔMBCS2022^ and H3^2003^ attached more abundantly than H5^ΔMBCS2005^. In bottlenose dolphins, H3^2003^ attached most abundantly (Table; Figure 2).

Overall, in the lower respiratory tract of seals and harbor porpoises, H5^ΔMBCS2022^ attached more abundantly than H5^ΔMBCS2005^. In bottlenose dolphins, H3^2003^ attached most abundantly, with only limited attachment of H5^ΔMBCS2022^ in the bronchiole and alveoli.

## Discussion

We describe the attachment patterns of HPAI H5N1 viruses in the respiratory tracts of common North Atlantic marine mammals. Our study revealed that avian H5 viruses attach abundantly to the upper respiratory tract of harbor seals and gray seals. In the lower respiratory tract of harbor seals, gray seals, and harbor porpoises, the recent H5N1 clade 2.3.4.4b virus attaches more abundantly than an H5N1 clade 2.1.3.2 virus from 2005.

The attachment pattern of HPAI H5N1 viruses to both the upper and lower respiratory tract tissues of North Atlantic marine mammals is in line with the detection of infectious virus or viral RNA in respiratory tract tissues of all included species (*21*,*23*,*24*,*26*). Unfortunately, little is known about the cell tropism of H5N1 viruses in vivo; pathological studies on the cell tropism are scarce, and tissues from infected marine mammals are often not representative for the acute phase of infection. However, virus antigen has been detected in bronchiolar and alveolar epithelial cells in harbor seals, which fits with the ability of H5N1 virus to attach to those cells (*21*). Abundant attachment to the upper respiratory tract of pinnipeds suggests that the species are highly susceptible to infection and that viruses can be transmitted among them. In the lower respiratory tract of harbor seals and gray seals, the HA of H5N1 clade 2.3.4.4b virus had the tendency to attach more abundantly than H5N1 clade 2.1.3.2 virus. That difference could contribute to the ability of clade 2.3.4.4b viruses to cause severe lower respiratory tract disease and fits with the high mortality rates reported in harbor and gray seals (*21*,*23*,*24*). High mortality rates associated with clade 2.3.4.4b virus infections also has been reported in sea lions and elephant seals (*3*,*12*,*44*). Whether the observed attachment pattern of clade 2.3.4.4b viruses in phylogenetically distinct pinniped species would be similar remains unknown. In both cetacean species tested, the clade 2.3.4.4b virus attached more abundantly to the respiratory tract than did clade 2.1.3.2 virus, but the attachment was overall lower than for both pinniped species. That finding is consistent with the individual cases of H5N1 virus infection in harbor porpoises, bottlenose dolphins, and other cetacean species (*8*,*26*–*28*), suggesting that cetaceans are also susceptible to infection. The ability of H5N1 viruses to attach to respiratory tissues of marine mammals is not unique; avian influenza viruses of subtypes H5N4 and H7N7 can also attach to tissues of the lower respiratory tract (*33*). However, the observed attachment pattern for HPAI H5N1 clade 2.3.4.4b viruses likely contributes to the high number of infections and the development of severe disease.

Several studies have shown that recent H5N1 clade 2.3.4.4b viruses, including bovine isolates, preferentially bind to α2,3-linked sialic acid receptors (*41*,*45*–*47*). The variability in attachment between the 2 H5N1 virus clades in our study are therefore likely not the result of a receptor switch to 2,6-linked sialic acid but potentially because of the amino acid differences in or close to the receptor-binding site, known to affect receptor specificity or affinity. However, the exact role of the individual amino acid positions remains to be investigated.

Both HPAI H5N1 viruses (either of clade 2.3.4.4b or clade 2.1.3.2) and H3N2 virus attach to olfactory mucosa in the nasal cavity of gray and harbor seals. Neurologic complications are regularly observed in marine mammals infected with H5 viruses, and virus can be detected in high titers in the brain (*19*,*21*,*23*,*24*,*28*). How H5 viruses enter the central nervous system remains unclear, but observations suggest that the viruses can enter the central nervous system via the olfactory nerve in seals, as observed in experimentally inoculated ferrets (*48*–*50*). However, HPAI H5N1 viruses can also invade the central nervous system in ceteceans, which lack a olfactory mucosa, so neuroinvasion likely could also occur via other cranial nerves or the hematogenous route (*28*).

In conclusion, our study highlights changes in the attachment pattern of a recent HPAI H5N1 clade 2.3.4.4b virus compared with H5N1 clade 2.1.3.2 virus from 2005 in the respiratory tracts of 4 marine mammal species that could lead to more efficient transmission and more severe disease. That finding, together with the recent increase in HPAI H5N1–associated deaths in marine mammals worldwide, emphasizes the need for increased avian influenza surveillance and research in such marine mammal species to limit illness and deaths and help protect both animal and human health.

Appendix Additional information for attachment pattern of avian influenza H5 clade 2.3.4.4b virus in respiratory tracts of marine mammals, North Atlantic Ocean.
