# Supplementary material for: Attachment Patterns of Avian Influenza H5 Clade 2.3.4.4b Virus in Respiratory Tracts of Marine Mammals, North Atlantic Ocean
Source: Emerg Infect Dis. 2025 Sep;31(9):1729–37. doi: 10.3201/eid3109.250499 (PMC12407193; doi:10.3201/eid3109.250499)
Supplement: Appendix — Additional information for attachment pattern of avian influenza H5 clade 2.3.4.4b virus in respiratory tracts of marine mammals, North Atlantic Ocean. [file 25-0499-Techapp-s1.pdf]

Article DOI: https://doi.org/10.3201/eid3109.250499

EID cannot ensure accessibility for supplementary materials supplied by authors. Readers who have difficulty accessing supplementary content should contact the authors for assistance.

Attachment Patterns of Avian Influenza H5 Clade 2.3.4.4b Virus in Respiratory Tracts of Marine Mammals, North Atlantic Ocean

Appendix

Marine Mammal HA Sequences for generating the consensus

We downloaded all available influenza A H5 HA nucleotide sequences and accompanying metadata from GISAID (https://www.gisaid.org) on 11 January 2025:

EPI_ISL_19313608: A/Antarctic Fur Seal/South Georgia and the South Sandwich Islands/128/2023 (A/H5N1)

EPI_ISL_19313608: A/Antarctic Fur Seal/South Georgia and the South Sandwich Islands/128/2023 (A/H5N1)

EPI_ISL_19313544: A/Southern Elephant Seal/South Georgia and the South Sandwich Islands/24/2023 (A/H5N1)

EPI_ISL_19313544: A/Southern Elephant Seal/South Georgia and the South Sandwich Islands/24/2023 (A/H5N1)

EPI_ISL_19215180: A/Otaria flavescens/Rio Grande do Sul/2165-SO/2023 (A/H5N1)

EPI_ISL_19215180: A/Otaria flavescens/Rio Grande do Sul/2165-SO/2023 (A/H5N1)

EPI_ISL_19215179: A/Otaria flavescens/Rio Grande do Sul/2148-N/2023 (A/H5N1)

EPI_ISL_19215179: A/Otaria flavescens/Rio Grande do Sul/2148-N/2023 (A/H5N1)

EPI_ISL_19070498: A/pinniped/Uruguay/P4_6923/2023 (A/H5N1)

EPI_ISL_19070492: A/pinniped/Uruguay/P8_8923/2023 (A/H5N1)

EPI_ISL_19070491: A/pinniped/Uruguay/P7_6923/2023 (A/H5N1)

EPI_ISL_19070490: A/pinniped/Uruguay/P6_6923/2023 (A/H5N1)

EPI_ISL_19070489: A/pinniped/Uruguay/P5_6923/2023 (A/H5N1)

EPI_ISL_19070486: A/pinniped/Uruguay/P18_14923/2023 (A/H5N1)

EPI_ISL_19070485: A/pinniped/Uruguay/P17_14923/2023 (A/H5N1)

EPI_ISL_19070484: A/pinniped/Uruguay/P15_14923/2023 (A/H5N1)

EPI_ISL_19070483: A/pinniped/Uruguay/P14_11923/2023 (A/H5N1)

EPI_ISL_19070482: A/pinniped/Uruguay/P13_11923/2023 (A/H5N1)

EPI_ISL_19070481: A/pinniped/Uruguay/P10_8923/2023 (A/H5N1)

EPI_ISL_18945320: A/South American fur seal/Argentina/RN-PB019/2023 (A/H5N1)

EPI_ISL_18945319: A/South American sea lion/Argentina/RN-PB007/2023 (A/H5N1)

EPI_ISL_18945316: A/South American sea lion/Argentina/RN-PB013/2023 (A/H5N1)

EPI_ISL_18777141: A/porpoise/Antofagasta/SJCEIRR-2465062/2023 (A/H5N1)

EPI_ISL_18777140: A/porpoise/Atacama/SJCEIRR-245355/2023 (A/H5N1)

EPI_ISL_18777139: A/dolphin/Maule/SJCEIRR-246026/2023 (A/H5N1)

EPI_ISL_18777138: A/dolphin/Nuble/SJCEIRR-2482441/2023 (A/H5N1)

EPI_ISL_18777129: A/porpoise/Antofagasta/SJCEIRR-2465061/2023 (A/H5N1)

EPI_ISL_18760069: A/sea lion/Valparaiso/SJCEIRR-2431361/2023 (A/H5N1)

EPI_ISL_18742221: A/Southern_Elephant_Seal/Jason_Harbour/141027/2023 (A/H5N1)

EPI_ISL_18742213: A/Southern_Elephant_Seal/Jason_Harbour/141078/2023 (A/H5N1)

EPI_ISL_18742212: A/Antarctic_Fur_Seal/Jason_Harbour/141037/2023 (A/H5N1)

EPI_ISL_18737559: A/harbor seal/Washington/23–025744–002/2023 (A/H5N1)

EPI_ISL_18737558: A/harbor seal/Washington/23–025744–001/2023 (A/H5N1)

EPI_ISL_18731638: A/harbor seal/Washington/23–027069–002/2023 (A/H5N1)

EPI_ISL_18731622: A/harbor seal/Washington/23–025991–001/2023 (A/H5N1)

EPI_ISL_18731621: A/harbor seal/Washington/23–026504–002/2023 (A/H5N1)

EPI_ISL_18731620: A/harbor seal/Washington/23–026504–001/2023 (A/H5N1)

EPI_ISL_18698754: A/Sea Lion/Argentina/3849–4/2023 (A/H5N1)

EPI_ISL_18311028: A/harbor seal/Washington/23–026504–002/2023 (A/H5N1)

EPI_ISL_18311027: A/harbor seal/Washington/23–026504–001/2023 (A/H5N1)

EPI_ISL_18311025: A/harbor seal/Washington/23–025991–001/2023 (A/H5N1)

EPI_ISL_18311024: A/harbor seal/Washington/23–025744–002/2023 (A/H5N1)

EPI_ISL_18311023: A/harbor seal/Washington/23–025744–001/2023 (A/H5N1)

EPI_ISL_18265431: A/dolphin/Peru/PIU-SER002/2022 (A/H5N1)

EPI_ISL_18265430: A/South American Sea Lion/Peru/LIM-SER036/2023 (A/H5N1)

EPI_ISL_18265423: A/south american sea lion/Peru/AQP-SER00B/2023 (A/H5N1)

EPI_ISL_18265422: A/south american sea lion/Peru/AQP-SER00R/2023 (A/H5N1)

EPI_ISL_18265321: A/south american sea lion/Peru/AQP-SER00K/2023 (A/H5N1)

EPI_ISL_18054510: A/sea lion/Peru/AQP-SER00R/2023 (A/H5N1)

EPI_ISL_18054509: A/sea lion/Peru/AQP-SER00K/2023 (A/H5N1)

EPI_ISL_18054508: A/sea lion/Peru/AQP-SER00B/2023 (A/H5N1)

EPI_ISL_18054503: A/dolphin/Peru/PIU-002/2022 (A/H5N1)

EPI_ISL_18054502: A/Sea Lion/Peru/LIM-SER036/2023 (A/H5N1)

EPI_ISL_17885975: A/sea lion/Tarapaca/240524–2/2023 (A/H5N1)

EPI_ISL_17777532: A/sea_lion/Peru/TAC-INS-011/2023 (A/H5N1)

EPI_ISL_17777531: A/sea_lion/Peru/TAC-INS-010/2023 (A/H5N1)

EPI_ISL_17672783: A/gray seal/Netherlands/30448/2023 (A/H5N1)

EPI_ISL_17672782: A/gray seal/Netherlands/302603/2023 (A/H5N1)

EPI_ISL_17672781: A/gray seal/Netherlands/302579/2023 (A/H5N1)

EPI_ISL_17465835: A/harbor_seal/Scotland/054948/2022 (A/H5N1)

EPI_ISL_17465834: A/common_dolphin/England/040499/2023 (A/H5N1)

EPI_ISL_17465833: A/common_dolphin/Wales/040498/2023 (A/H5N1)

EPI_ISL_17465832: A/harbor_porpoise/England/040497/2023 (A/H5N1)

EPI_ISL_17424627: A/harbor seal/Maine/MME-185-ns/2022 (A/H5N1)

EPI_ISL_17424626: A/harbor seal/Maine/MME-117-ns/2022 (A/H5N1)

EPI_ISL_17424625: A/harbor seal/Maine/MME-150-L/2022 (A/H5N1)

EPI_ISL_17424624: A/harbor seal/Maine/MME-185-L/2022 (A/H5N1)

EPI_ISL_17424622: A/harbor seal/Maine/MME-147-L/2022 (A/H5N1)

EPI_ISL_17167249: A/gray seal/Germany-SH/AI01973/2023 (A/H5N1)

EPI_ISL_16904554: A/Harbour_Seal/Scotland/162925/2022 (A/H5N1)

EPI_ISL_16904553: A/Harbour_Seal/Scotland/162919/2022 (A/H5N1)

EPI_ISL_16632542: A/harbor seal/Maine/22–025072–002/2022 (A/H5N1)

EPI_ISL_16632541: A/harbor seal/Maine/22–022528–008/2022 (A/H5N1)

EPI_ISL_16632540: A/harbor seal/Maine/22–022528–006/2022 (A/H5N1)

EPI_ISL_16632539: A/harbor seal/Maine/22–022528–003/2022 (A/H5N1)

EPI_ISL_16212269: A/bottlenose dolphin/Florida/UFTt2203/2022 (A/H5N1)

EPI_ISL_15078255: A/dolphin/Florida/22–025319–002/2022 (A/H5N1)

EPI_ISL_15069397: A/bottlenose dolphin/Florida/UFTt2203/2022 (A/H5N1)

EPI_ISL_14810369: A/porpoise /Sweden/SVA220712SZ0367/FB002184/O-2022 (A/H5N1)

EPI_ISL_14098924: A/harbor seal/Maine/22–020983–007/2022 (A/H5N1)

EPI_ISL_14098923: A/harbor seal/Maine/22–020983–006/2022 (A/H5N1)

EPI_ISL_14098922: A/gray seal/Maine/22–020983–003/2022 (A/H5N1)

EPI_ISL_14098921: A/harbor seal/Maine/22–020983–002/2022 (A/H5N1)

EPI_ISL_14098920: A/harbor seal/Maine/22–020983–001/2022 (A/H5N1)

EPI_ISL_14098919: A/harbor seal/Maine/22–020455–005/2022 (A/H5N1)

EPI_ISL_14098918: A/harbor seal/Maine/22–020455–004/2022 (A/H5N1)

EPI_ISL_14098917: A/harbor seal/Maine/22–020455–003/2022 (A/H5N1)

EPI_ISL_14098916: A/harbor seal/Maine/22–020455–002/2022 (A/H5N1)

EPI_ISL_14098915: A/harbor seal/Maine/22–020455–001/2022 (A/H5N1)

EPI_ISL_4805936: A/seal/Germany-SH/AI05379/2021 (A/H5N8)

EPI_ISL_4805852: A/seal/Germany-SH/AI05377/2021 (A/H5N8)

EPI_ISL_4804850: A/seal/Germany-SH/AI05373/2021 (A/H5N8)

EPI_ISL_2081528: A/seal/England/AVP-031141/2020 (A/H5N8)

EPI_ISL_1789695: A/Grey Seal/SVA210406SZ0066/KN001401/2021 (A/H5N8)

EPI_ISL_362127: A/Grey seal/361–13/BalticPL/16 (A/H5N8)

EPI_ISL_322984: A/Grey seal/361–10/BalticPL/16 (A/H5N8)

EPI_ISL_5729: A/Indonesia/5/2005 (A/H5N1)

EPI_ISL_16524246: A/Caspian gull/Netherlands/1/2023 (A/H5N1)

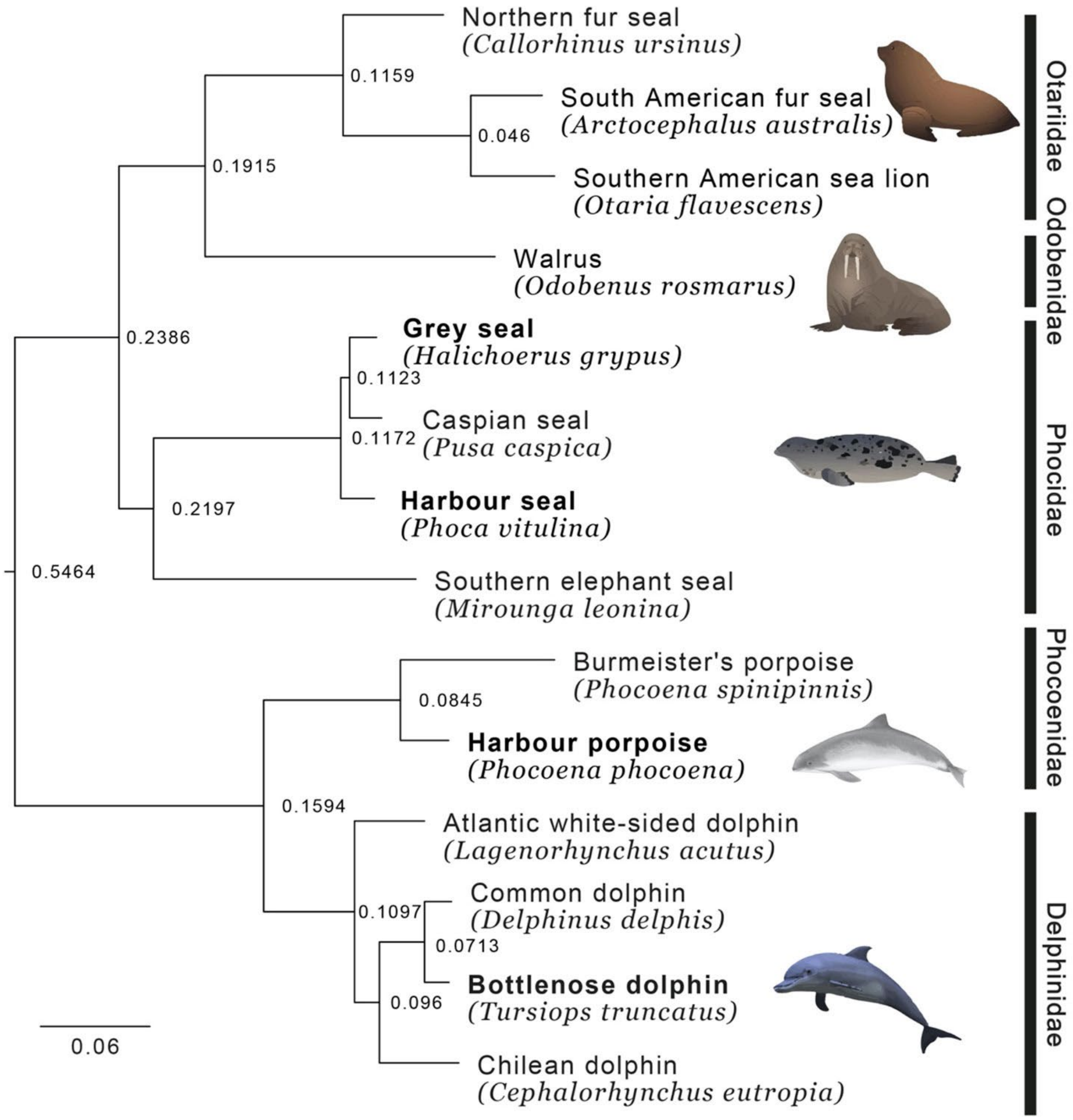

Appendix Figure 1. Phylogeny of marine mammal species affected worldwide by highly pathogenic avian influenza virus A (HPAI) H5N1 clade 2.3.3.4b from the panzootic (2022–2023). Maximum Likelihood tree constructed using RAxML-HPC2 on ACESS (8.2.12) with rapid bootstrapping run on XSDE (*1*), from DNA sequences available from NCBI taxonomy and Genbank.

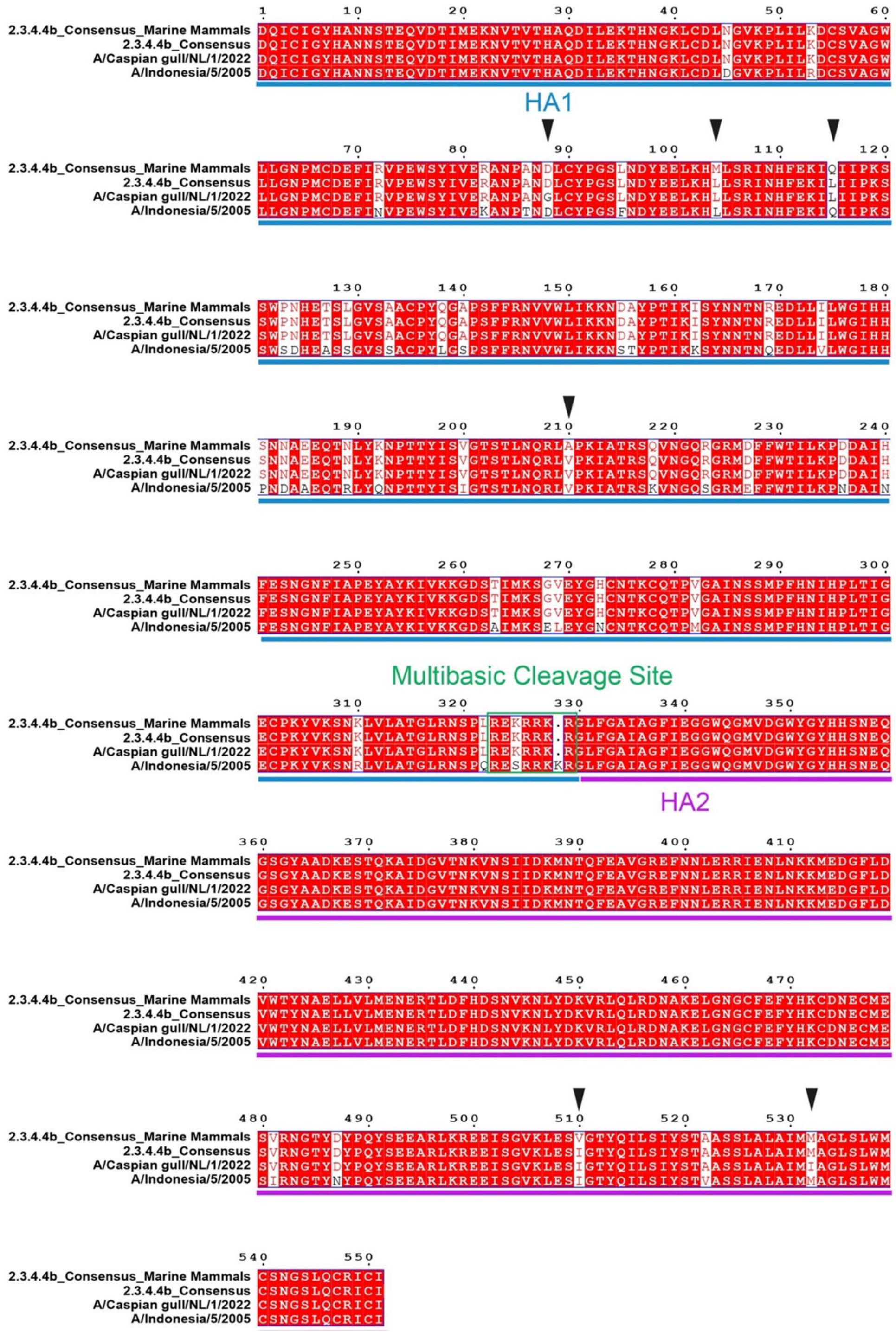

Appendix Figure 2. Multiple sequence alignment of H5N1 viruses. A multiple sequence alignment of the consensus sequence of all available HA of clade 2.3.4.4b in marine mammals and all available clade 2.3.4.4b viruses, A/Caspian gull/NL/2022 H5N1 ($H5^{\Delta MBCS2022}$) as previously described (L. Bauer et al., unpub. data, https://www.biorxiv.org/content/10.1101/2024.11.27.625596v1.full) and A/Indonesia/5/2005 H5N1 ($H5^{\Delta MBCS2005}$) was performed with Clustal OMEGA (2). The ESPRIPT3.0 (3) program was used to render sequence similarities. Differences in amino acids between 2.3.4.4b consensus marine mammals and A/Caspian gull/NL/2022 H5N1 aa are highlighted with black triangles.

References

1. Stamatakis A, Hoover P, Rougemont J. A rapid bootstrap algorithm for the RAxML Web servers. Syst Biol. 2008;57:758–71. PubMed https://doi.org/10.1080/10635150802429642

2. Madeira F, Madhusoodanan N, Lee J, Eusebi A, Niewielska A, Tivey ARN, et al. The EMBL-EBI Job Dispatcher sequence analysis tools framework in 2024. Nucleic Acids Res. 2024;52(W1):W521–5. PubMed https://doi.org/10.1093/nar/gkae241

3. Robert X, Gouet P. Deciphering key features in protein structures with the new ENDscript server. Nucleic Acids Res. 2014;42(Web Server issue):W320–4. PubMed https://doi.org/10.1093/nar/gku316
